# Durvalumab–Tremelimumab in Advanced Hepatocellular Carcinoma: Real‐World Data From the LOR‐HCC (Lombardy Real‐World HCC Group)

**DOI:** 10.1111/liv.70640

**Published:** 2026-04-16

**Authors:** Andrea Casadei‐Gardini, Lorenzo Canova, Federica Tosi, Silvia Camera, Elisa Bocchero, Pietro Pozzoni, Martina Torchio, Salvatore Corallo, Daniela Costantino, Annamaria Stagno, Giulia Grizzi, Rita Gengarle, Gianluca Tomasello, Nasim Ansarin, Mariangela Bruccoleri, Katia Bencardino, Mara Persano, Martina Pino, Alessia Riva, Sara Vavassori, Lorenzo Argiento, Chiara Mazzarelli, Massimo De Giorgio, Elena Rota Caremoli, Margherita Rimini, Tiziana Pressiani, Lorenza Rimassa, Massimo Iavarone

**Affiliations:** ^1^ Department of Oncology Vita‐Salute San Raffaele University, IRCCS San Raffaele Scientific Institute Hospital Milan Italy; ^2^ Division of Hepatology San Giuseppe Hospital, IRCCS MultiMedica Milan Italy; ^3^ The Falck Division of Medical Oncology, Niguarda Cancer Center, Grande Ospedale Metropolitano Niguarda Milan Italy; ^4^ Department of Biomedical Sciences Humanitas University Milan Italy; ^5^ Humanitas Cancer Center, IRCCS Humanitas Research Hospital Rozzano Italy; ^6^ Hepatology Unit, Department of Medicine, Alessandro Manzoni Hospital, ASST Lecco Lecco Italy; ^7^ U.O.C. Oncologia Medica, ASST Santi Paolo e Carlo, Ospedale San Carlo Borromeo Milan Italy; ^8^ SC Oncology Unit, IRCCS Foundation, San Matteo Hospital Pavia Italy; ^9^ Department of Organ Failure and Transplantation, Gastroenterology, Hepatology and Liver Transplantation Ospedale Papa Giovanni XXIII Bergamo Italy; ^10^ SC Medical Oncology, Fondazione IRCCS San Gerardo Dei Tintori Monza Italy; ^11^ Division of Medical Oncology ASST Cremona Cremona Italy; ^12^ Complex Medical Oncology Unit, ASST Mantova, Carlo Poma Hospital Mantova Italy; ^13^ Department of Oncology ASST Ospedale Maggiore di Crema Crema Italy; ^14^ Division of Gastroenterology and Hepatology Foundation IRCCS ca’ Granda Ospedale Maggiore Policlinico Milan Italy; ^15^ Medicina Ad Indirizzo Epatologico e Gastroenterologia, ASST Santi Paolo e Carlo, Ospedale San Paolo Milan Italy; ^16^ Hepatology & Gastroenterology, Grande Ospedale Metropolitano Niguarda Milan Italy; ^17^ Medical Oncology‐Ospedale Papa Giovanni XXIII Bergamo Italy; ^18^ CRC A. M. and A. Migliavacca Center for Liver Disease, Department of Pathophysiology and Transplantation University of Milan Milan Italy

## Abstract

**Background and Aim:**

Dual immune checkpoint blockade with tremelimumab plus durvalumab (STRIDE) is an established first‐line therapy for unresectable hepatocellular carcinoma (uHCC); however, real‐world evidence on its safety, toxicity kinetics and liver function dynamics remains limited. We aimed to evaluate the tolerability and on‐treatment changes in hepatic function and effectiveness in a multicentre cohort of patients treated with STRIDE in routine practice.

**Methods:**

We conducted a retrospective analysis of prospectively collected data from 115 consecutive patients with uHCC treated with STRIDE across 12 centres in Lombardy, Italy. Clinical, biochemical and radiological variables were recorded at baseline and throughout the therapy. Adverse events were graded per CTCAE v5.0, and Child–Pugh was used to assess hepatic functional evolution. The temporal toxicity patterns were evaluated using smoothed hazard functions. Progression‐free survival (PFS), overall survival (OS) and competing‐risk cumulative incidences of progression and death were examined.

**Results:**

The median follow‐up was 8.9 months. More than 80% of the adverse events occurred within the first 2 months, and severe toxicities were infrequent. Child–Pugh deterioration from class A to B occurred in 7.8% of patients from baseline to day 28, increasing to 12.1% by day 56. PFS was 5.7 months; the median OS was not reached, with OS rates of 78.2% at 6 months and 53.3% at 18 months. Progression was the predominant early event, with a cumulative incidence of 49.2% at 6 months.

**Conclusions:**

In routine clinical practice, STRIDE shows reproducible effectiveness and a manageable safety profile, with an early and stabilising incidence of adverse events. Dynamic assessment of liver function shows that hepatic function declines modestly but is still clinically meaningful in affected patients.

## Highlights

- Durvalumab plus tremelimumab treatment (STRIDE regimen) shows reproducible effectiveness and a manageable safety profile in real‐world cases of unresectable HCC.
- Most adverse events (AE) cluster early and then stabilise; over 80% of AE (i.e., fatigue, rash and itching) occur within the first 2 months, with infrequent severe toxicities. A modest, but significant, decline in liver function was observed.
- The STRIDE regimen achieved a median PFS of 5.7 months; median OS was not reached (78.2% at 6 months, 53.3% at 18 months).
- This study highlights the critical role of the liver functional reserve in treatment outcomes and the need for early toxicity monitoring.

**Abbreviations**
AE: Adverse Event
AFP: Alpha‐Foetoprotein
ALBI: Albumin–Bilirubin Score
ALT: Alanine Aminotransferase
AST: Aspartate Aminotransferase
BCLC: Barcelona Clinic Liver Cancer
CI: Confidence Interval
CP: Child–Pugh
CR: Complete Response
CTCAE: Common Terminology Criteria for Adverse Events
DC: Disease Control
DCR: Disease Control Rate
ECOG‐PS: Eastern Cooperative Oncology Group Performance Status
ESMO: European Society for Medical Oncology
HCC: Hepatocellular Carcinoma
HR: Hazard Ratio
IQR: Interquartile Range
irAE: Immune‐Related Adverse Event
LOR‐HCC: Durvalumab–Tremelimumab Lombardy Real‐World Study
mRECIST: Modified Response Evaluation Criteria in Solid Tumours
NE: Not Evaluable
OR: Odds Ratio
OS: Overall Survival
PD: Progressive Disease
PFS: Progression‐Free Survival
PR: Partial Response
RECIST: Response Evaluation Criteria in Solid Tumours
RFA: Radiofrequency Ablation
ROC: Receiver Operating Characteristic
SBRT: Stereotactic Body Radiotherapy
SD: Stable Disease
SIRT: Selective Internal Radiotherapy (Yttrium‐90 Radioembolisation)
STRIDE: Single Tremelimumab Regular Interval Durvalumab
TACE/TAE: Transarterial Chemoembolisation/Transarterial Embolisation
TARE: Transarterial Radioembolisation
TTP: Time to Progression
UGE: Upper Gastrointestinal Endoscopy

## 1 Introduction

Immune checkpoint inhibitors have reshaped the treatment landscape of unresectable hepatocellular carcinoma (uHCC), a disease historically characterised by limited systemic options and poor long‐term outcomes [1, 2, 3]. Following the results of IMbrave150, the combination of atezolizumab plus bevacizumab became the first immunotherapy‐based standard of care in the first‐line setting, demonstrating a clear survival advantage over sorafenib in patients with preserved liver function [4, 5]. Subsequent phase III trials further expanded the number of active regimens and led to the approval of the STRIDE regimen, which combines a single priming dose of tremelimumab with durvalumab and provides durable survival benefit without the need for anti‐angiogenic therapy [6, 7, 8]. More recently, the CheckMate 9DW phase III study demonstrated the benefit of the combination of nivolumab and ipilimumab over lenvatinib/sorafenib in the same setting [9]. Nevertheless, most of this evidence originates from randomised controlled trials that enrolled highly selected patients, typically restricted to Child–Pugh (CP) A cirrhosis, Eastern Cooperative Oncology Group performance status (ECOG‐PS) 0–1 and absence of high‐risk portal vein invasion, raising questions about the generalisability of these results to the broader population seen in clinical practice.

Real‐world data with atezolizumab plus bevacizumab have begun to address this gap [10, 11, 12]. A international multicentre study, including patients with both CP A and B cirrhosis, showed that the combination can be delivered beyond the strict trial‐eligible population, with a safety profile that remains acceptable but is clearly modulated by liver reserve, as patients with CP‐B cirrhosis experienced higher rates of hepatic decompensation and treatment modification compared with CP‐A [10]. Building on this experience, a subsequent analysis on the same dataset demonstrated that hepatic decompensation is the main driver of mortality in patients with uHCC treated with atezolizumab plus bevacizumab, and that optimisation of the underlying liver disease mitigates this risk [12]. Together, these studies highlight how, in immunotherapy‐treated uHCC, survival is determined by the intertwined trajectories of tumour control and liver function, and they underscore the need for careful characterisation of liver‐related events under treatment [13, 14].

The same group recently extended this paradigm to durvalumab‐based regimens through an international multicentre real‐world analysis of patients treated with durvalumab plus or minus tremelimumab in routine practice (DT‐real). The study confirmed that the STRIDE regimen achieves reproducible survival outcomes outside clinical trials, with a median overall survival (OS) of approximately 20 months in the overall cohort and around 23 months in patients who fulfilled the key HIMALAYA eligibility criteria [15]. Importantly, this real‐world study reinforced the prognostic role of liver function by identifying hepatic decompensation, together with macrovascular invasion, as an independent determinant of mortality, and showed that about 10% of patients experienced decompensation within the first year of therapy [9]. Disease control at radiological assessment translated into substantial long‐term survival, echoing the landmark findings of HIMALAYA, further emphasising that durable benefit from dual checkpoint blockade occurs primarily in patients who can maintain adequate hepatic reserve throughout treatment. Beyond survival, the temporal behaviour of immune‐related adverse events (irAEs) has emerged as another key dimension of treatment evaluation [15]. A dedicated analysis of immune‐related liver injury across HCC and non‐HCC indications showed that hepatic toxicity under checkpoint inhibition tends to occur earlier and more frequently in patients with HCC, yet does not inevitably compromise treatment continuation or OS when adequately recognised and managed [16].

Within this evolving context, real‐world evidence remains essential to clarify the safety and hepatic tolerance of the STRIDE regimen beyond the trial‐selected populations. In this study, we report the preliminary results of an ongoing multicentre experience with durvalumab plus tremelimumab (LOR‐HCC), focusing on the temporal pattern of Adverse Event (AEs) and the behaviour of liver function during treatment.

## 2 Methods

### 2.1 Study Design

The LOR‐HCC study was a retrospective analysis of prospectively collected data from multiple hepatology and oncology centres in Lombardy, Italy. The database was specifically designed to systematically capture clinical, laboratory and radiological information from patients treated with the STRIDE regimen, which consists of a single priming dose of tremelimumab followed by durvalumab, in routine clinical practice starting in 2022. For the present analysis, all consecutive patients treated with STRIDE up to the data‐lock date of October 01, 2025, were included.

Eligible patients were diagnosed with uHCC according to international guidelines and received STRIDE as the first‐line systemic therapy. Cirrhosis was defined based on either histopathological confirmation or imaging findings (nodular liver surface, splenomegaly or varices) or clinical features of portal hypertension. Patients with compensated or mildly decompensated cirrhosis (CP score B7) were included to reflect real‐world clinical practice. The exclusion criteria were the absence of baseline imaging, clinical and laboratory data, incomplete follow‐up data, or discontinuation of treatment before the first scheduled radiological assessment for nonclinical reasons.

In routine clinical practice across participating centres, the choice of STRIDE as first‐line therapy reflected physician clinical judgment and institutional experience following regulatory approval, rather than predefined selection criteria, and the specific reasons for choosing STRIDE over other approved regimens were not systematically recorded in the database.

The primary objective of this study was to characterise the safety profile of STRIDE in a real‐world population, with a particular focus on the temporal pattern of AEs, including irAEs and liver‐related toxicities. A key objective of this study was to evaluate the clinical impact of treatment on hepatic functional reserve. Hepatic functional reserve was longitudinally assessed throughout the treatment using dynamic measures, including CP score evolution, biochemical parameters and the occurrence of cirrhosis‐related clinical decompensation events.

Secondary objectives included the assessment of anti‐tumour activity based on radiological outcomes and the evaluation of survival endpoints.

Radiological evaluations were performed approximately every 8–12 weeks according to institutional practice.

Follow‐up started at the date of the first STRIDE administration and continued until death, last available clinical contact, or initiation of subsequent systemic therapy, whichever occurred first. Adverse events and laboratory parameters were assessed at each treatment visit and during unscheduled evaluations, when clinically indicated.

This study was conducted in accordance with the principles of the Declaration of Helsinki. Data collection and sharing were approved by the ethics committee of the coordinating centre and the local institutional review boards at each participating site.

### 2.2 Statistical Methods

Baseline variables extracted from the shared database included demographic characteristics, liver disease aetiology, cirrhosis status, CP and ALBI scores, ECOG‐PS, laboratory parameters, and tumour burden features (number and size of HCC lesions, macrovascular invasion, extrahepatic spread and Barcelona Clinic Liver Cancer [BCLC] stage). Treatment‐related variables included the dates of drug administration, treatment interruptions or discontinuations, and corticosteroid use for toxicity management.

The baseline characteristics were summarised using descriptive statistics. Categorical variables were reported as counts and percentages, while continuous variables were expressed as mean ± standard deviation or median and interquartile range (IQR), as appropriate. Comparisons between responders and non‐responders were performed using χ^2^ or Fisher's exact test for categorical variables and Student's *t*‐test or Mann–Whitney U test for continuous variables. For categorical variables, the proportion of responders in each category is also reported.

OS was defined as the time from treatment initiation to death from any cause, and progression‐free survival (PFS) was defined as the time from treatment initiation to radiological progression or death. Patients without adverse events were censored at the last follow‐up visit. Survival curves were estimated using the Kaplan–Meier method and compared using the log‐rank test. Landmark survival rates are reported at predefined time points. Hazard ratios (HRs) with 95% confidence intervals (CIs) were estimated using Cox proportional hazards models; variables with *p* < 0.10 in univariable analysis were entered into multivariable models. The proportional hazard assumption was assessed using Schoenfeld residuals.

Continuous laboratory variables without established clinical cut‐offs were evaluated using ROC curve analysis, with death as the outcome, and optimal thresholds were identified using the Youden index. The variables were subsequently dichotomised. Alpha‐foetoprotein (AFP) level was analysed as a continuous variable and dichotomised at 400 ng/mL. Objective response at first radiological evaluation was assessed in routine clinical practice according to RECIST v1.1, based on whole tumour measurements on locally performed contrast‐enhanced CT or MRI scans, and subsequently analysed using logistic regression models [17].

Adverse events were graded according to the CTCAE v5.0. Time‐to‐toxicity was defined as the interval between treatment initiation and the first occurrence of each AE. Temporal toxicity patterns were analysed using kernel density estimation to derive smoothed hazard functions expressed as events per patient‐month. Hazard functions were visualised as individual curves and heatmaps (0–15 months) separately for all‐grade and grade ≥ 3 AEs, with normalised intensities to facilitate comparison.

All statistical tests were two‐sided, with *p* < 0.05 considered statistically significant. Analyses were performed using the R software version 4.5.1.

## 3 Results

### 3.1 Patient Characteristics

A total of 115 patients were included in the analysis, with a median follow‐up of 8.9 months (range 7.3–18.1). Baseline patient characteristics are summarised in Table 1, which shows a predominantly elderly male population with preserved liver function and advanced‐stage disease. Upper gastrointestinal endoscopy (UGE) was performed in 71.3% of patients within 6 months prior to STRIDE initiation, while 10.4% had been performed more than 6 months before the start of treatment, and 13.9% of patients did not undergo UGE before initiating therapy. Among the 28.7% of patients with oesophageal varices at baseline, 48.5% had F1 varices and 48.5% had F2 varices, whereas high‐grade varices (F3) were not observed. Gastric varices were less frequent, being present in 6.1% of the patients with baseline oesophageal varices.

**TABLE 1:** Baseline characteristics of patients included in the study (*n* = 115).

Characteristic	Category/value	*N* (%) or median (IQR)
Demographics		
Age	≥ 70 years	79 (68.7)
Sex	Male	98 (85.2)
Aetiology		
	ALD	20 (17.4)
	HBV	24 (20.9)
	HCV	43 (37.4)
	MASLD	34 (29.6)
	Met‐ALD	14 (12.2)
Liver disease		
	Cirrhosis	94 (81.7)
	Child–Pugh class A	100 (87.0)
	Child–Pugh class B	15 (13.0)
	Ascites	12 (10.5)
	Hepatic encephalopathy	2 (1.7)
Laboratory values (continuous)		
ALT, U/L		33.0 (23.5–50.0)
AST, U/L		44.0 (29.5–67.5)
Total bilirubin, mg/dL		0.85 (0.60–1.20)
Albumin, g/dL		3.80 (3.50–4.20)
Creatinine, mg/dL		0.98 (0.77–1.20)
AFP, ng/mL		30.9 (5.7–396.0)
Platelets, ×10^3^/μL		194 (144–252)
Sodium, mmol/L		139 (136–142)
Laboratory values (categorical)		
ALT ≥ 41 U/L		40 (34.8)
AST ≥ 52 U/L		37 (32.2)
Bilirubin ≥ 0.95 mg/dL		41 (35.7)
Albumin < 3.5 g/dL		27 (23.9)
Creatinine ≥ 1.72 mg/dL		8 (7.0)
AFP ≥ 400 ng/mL		25 (23.8)
Platelets ≤ 194 000/μL		87 (75.7)
Leukocytes ≤ 4660/μL		34 (29.6)
Neutrophils ≥ 3259/μL		71 (61.7)
Lymphocytes ≤ 800/μL		26 (22.6)
Monocytes ≥ 490/μL		73 (63.5)
Basophils > 20/μL		64 (55.7)
Eosinophils ≥ 120/μL		62 (53.9)
Haemoglobin ≤ 9.9 g/dL		7 (6.1)
Sodium < 135 mmol/L		11 (10.7)
Tumour characteristics		
BCLC stage	B	37 (32.2)
	C	78 (67.8)
Extrahepatic spread	Yes	46 (40.0)
Neoplastic portal vein invasion	Vp1–Vp3	31 (26.9)
	Vp4	6 (5.9)
Performance status		
ECOG PS	0	47 (40.9)
	1	62 (53.9)
	2	6 (5.2)

Abbreviations: AFP, alfa‐fetoprotein; ALD, alcoholic liver disease; ALT, alanine aminotransferase; AST, aspartate aminotransferase; BCLC, Barcelona Clinic Liver Cancer; ECOG PS, Eastern Cooperative Oncology Group performance status; HBV, hepatitis B virus; HCV, hepatitis C virus; MASDL, metabolic dysfunction‐associated steatotic liver disease; Met‐ALD, metabolic dysfunction‐associated alcohol‐associated liver disease.

### 3.2 Safety and Toxicity Profile

**FIGURE 1:**
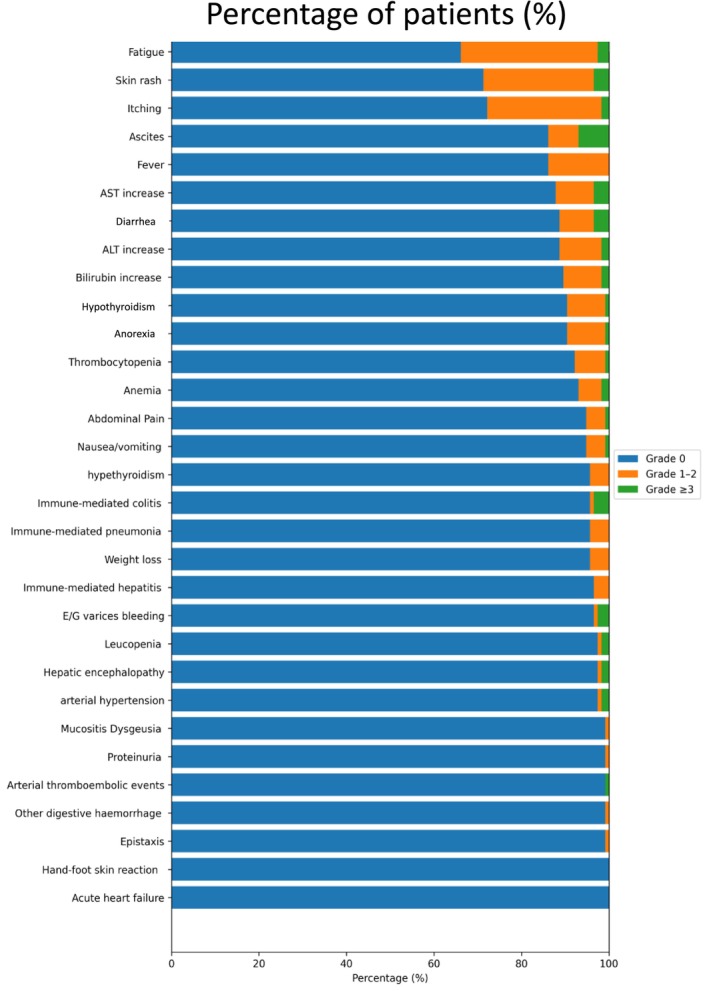
Distribution of adverse events. Percentage of patients experiencing treatment‐related adverse events during STRIDE therapy, categorised according to the highest CTCAE grade observed (grade 1, grade 2 and grade ≥ 3).

AEs were reported in 92 (80.0%) patients. The most frequent all‐grade AEs were fatigue (37.4%), rashes (30.4%) and itching (29.6%). Other relevant AEs included increased AST (18.0%), ALT (17.1%), ascites (16.2%), and fever (13.9%). Severe (G ≥ 3) AEs were infrequent, occurring in less than 8% of the patients; the most common AEs were AST increase (5.4%), ALT increase (4.5%), ascites (7.2%), fatigue (4.3%) and diarrhoea (3.5%). IrAEs were rare, with grade ≥ 3 colitis (3.6%), hepatitis (1.8%) and pneumonia (0.9%) (Figure 1 and Table S1).

Hazard function analysis demonstrated that most AEs clustered early, with > 80% of all events, and nearly all grade ≥ 3 events peaked within the first 1–2 months of treatment. Early peaks were noted for fatigue (0.6 months), anorexia (0.4), diarrhoea (0.7), fever (0.1), rash (0.7), pruritus (0.7) and nausea/vomiting (0.6). Biochemical alterations, such as AST and ALT increased peaked at ~1 month, while immune‐mediated hepatitis peaked at 0.8 months. Haematological events occurred slightly later: anaemia peaked at 1.6 months, thrombocytopenia at 1.8 months and leukopenia at 3.4 months. Later events were infrequent but included hypothyroidism (2.4 months), hyperthyroidism (2.1 months), arterial thromboembolic events (2.8 months), hepatic encephalopathy (3.4 months), arterial hypertension (5.6 months) and proteinuria (9.8 months) (Figure S1).

Bleeding events occurred in four patients overall, graded as 2 and 3 in one patient each, and 4 in two patients. Among the patients who experienced oesophageal variceal bleeding, three had F2 varices at baseline upper endoscopy before treatment initiation.

**FIGURE 2:**
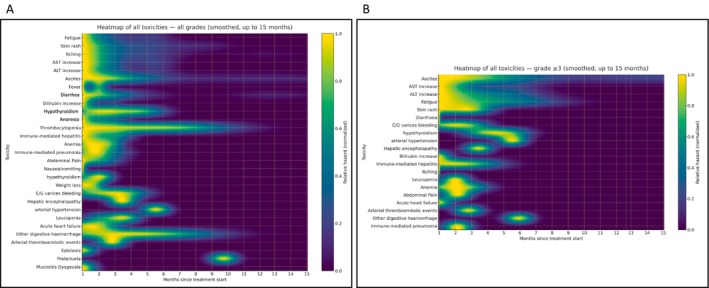
(A) Heatmap of adverse events by time since treatment initiation (0–15 months). Rows represent individual toxicities, and colour intensity indicates relative hazard (normalised). Vertical lines denote monthly intervals. (B) Heatmap of grade ≥ 3 adverse events during the first 15 months. Rows represent individual toxicities, and colour intensity indicates normalised hazard. Vertical lines denote monthly intervals. Toxicities are ordered by the overall incidence of grade ≥ 3 events.

Heatmaps summarising the hazard of all‐grade (Figure 2A) and grade ≥ 3 AEs (Figure 2B) confirmed the clustering of early toxicities and provided a visual overview of temporal toxicity dynamics.

Temporary interruption of immunotherapy was required in 37 (32.2%) patients. The leading causes were categorised as infectious complications (6.9%), dermatologic toxicities (5.2%), hepatic toxicity (1.7%), renal toxicity (1.7%), gastrointestinal toxicity (1.7%) and variceal or hemorrhagic events (1.7%). The remaining 13.0% were due to other reasons.

Given the observed frequency of AEs, we examined whether early toxicities were associated with treatment efficacy and survival outcomes. Analyses were restricted to early AEs occurring in at least five patients (≥ 4% of the cohort) within the first 60 days of treatment initiation. For each eligible AE, we assessed the association with radiological response (complete response, CR or partial response, PR vs. stable disease, SD or progressive disease, PD) using univariable logistic regression and evaluated PFS and OS with landmark Cox models at 60 days to minimise immortal‐time bias.

Several early AEs reached the predefined frequency threshold for analysis, the most common being aminotransferase increase and itching. Both AST and ALT elevations within 60 days were significantly associated with a higher probability of radiological response (OR, 5.66; 95% CI, 1.47–21.8; *p* = 0.012; 4.33; 95% CI, 1.56–8.75; *p* = 0.045 respectively), whereas early itching showed a non‐significant trend toward improved response (OR, 2.50; 95% CI, 0.90–6.94; *p* = 0.079). No other early AE was found to be statistically significant. In landmark analyses for PFS, early AST increase was associated with an HR of 0.92 (95% CI, 0.41–2.05; *p* = 0.84), ALT increase with an HR of 0.88 (95% CI, 0.38–2.04; *p* = 0.77) and pruritus (HR, 0.95; 95% CI, 0.48–1.90; *p* = 0.89). For OS, the corresponding HRs were 1.03 (95% CI, 0.45–2.36; *p* = 0.95) for AST increase, 0.97 (95% CI, 0.40–2.33; *p* = 0.94) for ALT increase and 0.90 (95% CI, 0.39–2.05; *p* = 0.80) for pruritus. Other early AEs with ≥ 5 events yielded nonsignificant results.

### 3.3 Longitudinal Changes in Liver Function

**FIGURE 3:**
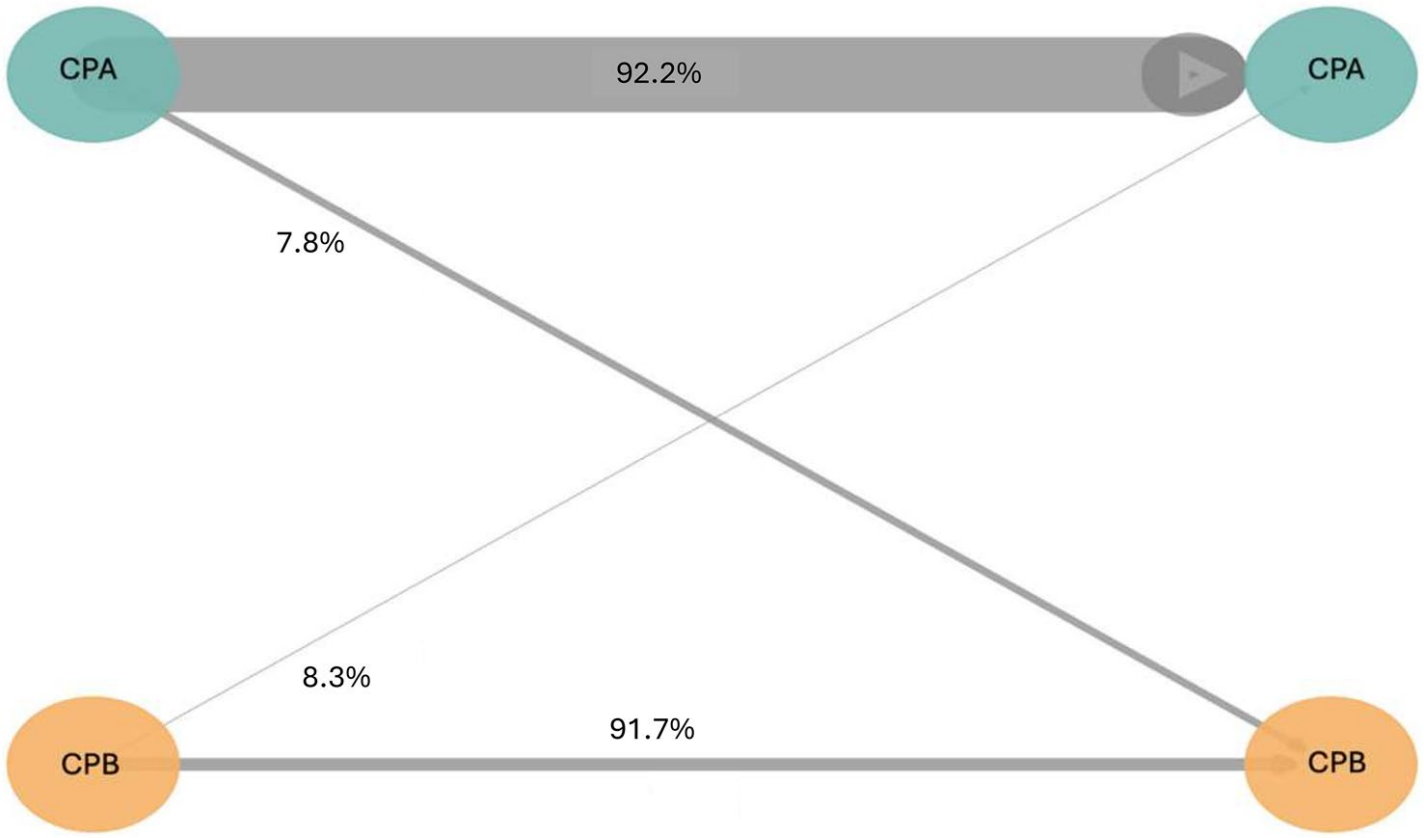
Child‐Pugh evolution analysis during first‐line treatment (STRIDE).

At the first evaluation (T1; 28 days after treatment initiation), 92.2% of patients with baseline CP‐A remained stable in class A, while 7.8% deteriorated to class B. Among patients with baseline CP‐B, 90.9% remained in class B and 9.1% improved to class A (Figure 3).

At the second evaluation (T2; 56 days after treatment initiation), 91.7% of patients with CP‐A at 28 days after treatment initiation remained stable, while 8.3% deteriorated to class B. Conversely, 94.7% of patients with CP‐B at 28 days after treatment initiation remained in class B, with a 5.3% improvement in class A.

Over the full course (T0–T2), 87.9% of patients with baseline CP‐A remained stable, while 12.1% deteriorated to class B; no patient with baseline CP‐B improved to class A.

Among the 95 patients with available data at both baseline and 28 days after treatment initiation, the mean ALBI score was −3.27 ± 0.48 at baseline and −2.74 ± 1.08 at 28 days after treatment initiation (*p* < 0.0001). When considering ALBI as a categorical variable, in 93% of patients, liver function was classified as ALBI grade 1 and in 7% as ALBI > 1 at baseline. At T1, 27% of patients with liver function initially classified as ALBI 1 shifted to ALBI > 1, whereas 71% of those with liver function initially categorised as ALBI > 1 improved to ALBI 1. Overall, the distribution of ALBI categories changed significantly over time (*p* = 0.0003).

Given the central role of liver functional reserve in advanced HCC, clinically overt hepatic decompensation events were additionally analysed as independent outcomes. During follow‐up, new or worsening ascites occurred in 20/115 patients (17.4%), hepatic encephalopathy in 3/115 (2.6%), variceal bleeding in 4/115 (3.5%) and bilirubin increase in 12/115 (10.4%). Treatment discontinuation attributed to liver decompensation occurred in 6/115 patients (5.2%), and 4/115 deaths (3.5%) were attributed to liver decompensation. Overall, 32/115 patients (27.8%) experienced at least one hepatic decompensation event (composite endpoint).

Using a competing‐risk framework with death as a competing event, the cumulative incidence of the composite hepatic decompensation endpoint was 14.4% at 3 months, 17.4% at 6 months and 20.8% at 12 months. When considering hepatic decompensation alongside progression and death as mutually competing first events, the 6‐month cumulative incidence was 16.4% for hepatic decompensation, 38.3% for radiological progression and 7.4% for death.

In cause‐specific Cox models, baseline Child–Pugh B7–9 (vs A5–6) was associated with a significantly higher risk of hepatic decompensation (HR 3.70, 95% CI 1.41–9.68; *p* = 0.0077), as was baseline ALBI (HR 2.87, 95% CI 1.25–6.62; *p* = 0.0132).

### 3.4 Effectiveness

**FIGURE 4:**
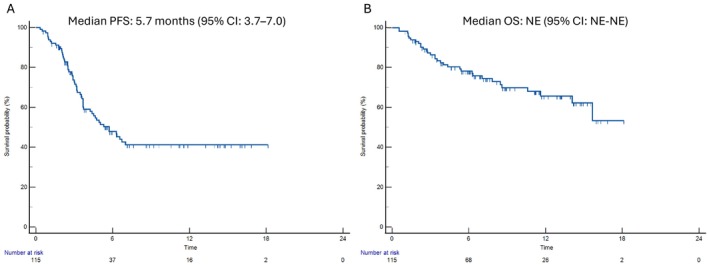
Kaplan–Meier estimates of progression‐free survival (A) and overall survival (B) in the overall study population (*n* = 115).

The median PFS was 5.7 months (95% CI 3.7–7.0) (Figure 4A), whereas the median OS was not reached at the time of analysis (Figure 4B). Kaplan–Meier estimates showed that 72.7% of patients remained progression‐free at 3 months, 47.9% at 6 months and 41.2% at 9 months, with stabilisation at 41.2% at 12, 15 and 18 months. OS rates were 86.3% at 3 months, 78.2% at 6 months, 69.8% at 9 months, 65.7% at 12 months, 62.2% at 15 months and 53.3% at 18 months.

Conditional survival analyses further highlighted the prognostic dynamics over time. Among the patients alive at 6 months, the probability of remaining alive at 15 and 18 months was 79.5% and 68.2% respectively. Similarly, among patients who were progression‐free at 4 months, the probability of remaining progression‐free at 12, 15 and 18 months was 63.9%. These findings indicate that, once patients overcome the initial risk period, the likelihood of maintaining long‐term benefits remains substantial.

**FIGURE 5:**
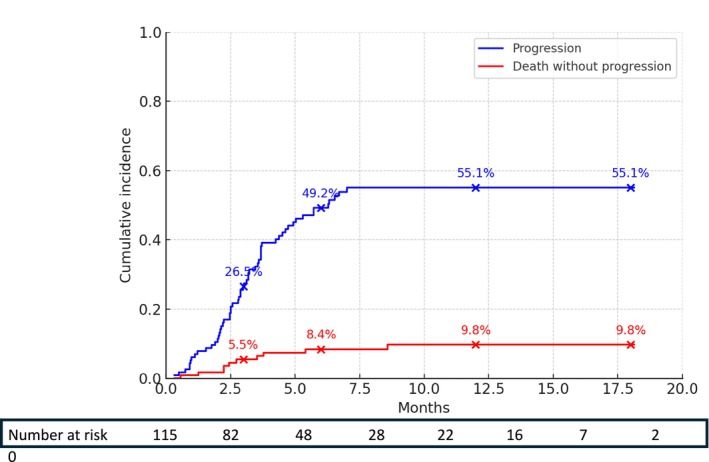
Cumulative incidence functions for radiological progression and death without documented progression.

In cumulative incidence analyses considering progression and death as competing events, disease progression was the predominant event across the follow‐up period. At 3 months, the cumulative incidence of progression was 26.5%, compared to 5.5% for death without progression. At 6 months, these values were 49.2% and 8.4% respectively; at 12 months, 55.1% and 9.8% respectively; and at 18 months, 55.1% and 9.8% respectively (Figure 5).

Of the 101 patients evaluated at the first radiological assessment, the best response was CR in 3.0%, PR in 21.8%, SD in 32.7% and PD in 42.5% for an overall disease control rate of 57.4%.

Patients achieving an objective radiological response (CR/PR) showed better survival compared to non‐responders. OS was not reached in responders versus 15.6 months (95% CI, 11.6–15.6) in non‐responders (HR 0.33, 95% CI 0.14–0.76; *p* = 0.0093) (Figure S2A). Similarly, PFS was not reached in responders versus 3.6 months (95% CI 3.0–4.6) in non‐responders (HR 0.24, 95% CI 0.14–0.42; *p* < 0.0001) (Figure S2B).

Patients with disease control (DC) had better survival than patients with PD, with the best radiological response. OS was not reached in the DC group versus 11.6 months (95% CI 4.0–15.6) in the PD group (HR 0.18, 95% CI 0.08–0.42; *p* = 0.0001) (Figure S2C). Similarly, PFS was not reached in the DC group versus 2.5 months (95% CI 2.0–7.0) in the PD group (HR 0.03, 95% CI 0.01–0.06; *p* < 0.0001) (Figure S2D). No significant associations were identified between the baseline patient characteristics and radiological responses, as shown in Figure S3.

Using a predefined 8‐week landmark analysis to minimise immortal time bias, patients achieving an objective radiological response (CR/PR) showed significantly improved survival compared with non‐responders. In the landmark‐restricted cohort, overall survival was significantly longer in responders (HR 0.19, 95% CI 0.04–0.80; *p* = 0.024) and progression‐free survival was also significantly improved (HR 0.11, 95% CI 0.03–0.35; *p* = 0.0002).

Using the same 8‐week landmark approach, patients achieving disease control (CR/PR/SD) showed significantly improved outcomes compared with those with progressive disease (PD) as the best radiological response. Disease control was associated with a significantly longer overall survival (HR 0.36, 95% CI 0.16–0.79; *p* = 0.011) and progression‐free survival (HR 0.10, 95% CI 0.05–0.20; *p* < 0.000001).

### 3.5 Prognostic Factors for Survival

At univariable Cox regression analysis, worse OS was significantly associated with elevated AST (> 52 U/L; HR 4.85, 95% CI 2.19–10.7, *p* = 0.0001), elevated ALT (> 41 U/L; HR 2.77, 95% CI 1.33–5.78, *p* = 0.0063), elevated bilirubin (> 0.95 mg/dL; HR 2.26, 95% CI 1.09–4.69, *p* = 0.028), ECOGPS ≥ 1 (HR 2.49, 95% CI 1.25–4.97, *p* = 0.0032), low albumin (< 3.5 g/dL; HR 0.22, 95% CI 0.09–0.54, *p* = 0.0011), hyponatraemia (< 135 mmol/L; HR 0.21, 95% CI 0.06–0.77, *p* = 0.018) and advanced tumour stage (BCLC C vs. B; HR 2.11, 95% CI 1.03–4.35, *p* = 0.041). Female sex was associated with a significantly lower risk of death (HR 0.18, 95% CI 0.05–0.57; *p* = 0.0039).

In the multivariable Cox regression model, three independent prognostic factors were confirmed: AST > 52 U/L (HR 3.01, 95% CI 1.22–7.40, *p* = 0.016), ECOG‐PS 1–2 (HR 2.95, 95% CI 1.17–7.40, *p* = 0.020) and female sex (HR 0.35, 95% CI 0.14–0.90, *p* = 0.028) (Table S2).

In univariable analysis, shorter PFS was significantly associated with elevated AST levels (> 52 U/L; HR 2.40, 95% CI 1.29–4.46, *p* = 0.0056), CP class B compared with class A (HR 4.01, 95% CI 1.51–10.7, *p* = 0.0054) and impaired PS, with ECOG‐PS > 0 associated with a higher risk of progression compared with ECOG‐PS 0 (HR 2.15, 95% CI 1.26–3.65, *p* = 0.02).

In the multivariable model, AST > 52 U/L (HR 2.22, 95% CI 1.28–3.87, *p* = 0.0046), ECOG‐PS 1 (HR 2.38, 95% CI 1.32–4.29, *p* = 0.0039), ECOG‐PS > 0 (HR 1.45, 95% CI 1.03–3.56, *p* = 0.0039) and CP‐B (HR 2.20, 95% CI 1.08–4.46, *p* = 0.028) remained independently associated with shorter PFS (Table S3).

## 4 Discussion

These preliminary analyses of our multicentre study indicate that the STRIDE regimen maintains a meaningful anti‐tumour activity and manageable safety profile in a real‐world cohort that reflects the heterogeneity of clinical practice more closely than those of controlled trials. The early and sustained disease control observed in our population, together with encouraging survival estimates despite limited follow‐up, reinforces the therapeutic relevance of dual checkpoint inhibition in advanced HCC. Conditional survival analyses further contextualise these findings by showing that patients who remain progression‐free or alive during the early treatment window experience a substantial improvement in their subsequent prognostic trajectory, supporting the clinical relevance of early disease control as an inflection point for long‐term outcomes while acknowledging the limitations of the relatively short follow‐up period. Furthermore, our findings highlight the central role of liver functional reserves, both at baseline and throughout treatment, in shaping clinical outcomes, underscoring how the biology of cirrhosis continues to modulate patient trajectories even in the era of immunotherapy [12, 13, 14].

Our observations extend previous clinical trials and contemporary real‐world studies examining programmed cell death protein 1/programmed death‐ligand 1 (PD‐1/PD‐L1)‐based regimens. The disease control rate and early progression dynamics in our cohort mirror the reproducible efficacy reported in large registries, including the DT‐real study, where real‐world outcomes were aligned closely with those achieved in the HIMALAYA population [6, 8, 15]. The convergence of results across cohorts differing in geography, practice patterns and baseline frailty suggests that the activity of STRIDE is robust and not limited to strictly selected populations. At the same time, our detailed temporal characterisation of AEs adds important complementary insight to existing literature [18]. The concentration of toxicities in the initial months of therapy, followed by a rapid decline in incidence, mirrors patterns described for immune‐related toxicity across HCC immunotherapy studies, but our hazard‐based depiction provides a more granular understanding of the critical early window during which liver vulnerability is greatest [15].

The implications of these findings on the biology of advanced HCC are notable. The early predominance of AEs, particularly those involving the hepatic function, reinforces the concept that cirrhosis remains a principal determinant of treatment tolerance. Even modest CP deterioration influenced survival trajectories, highlighting how immunotherapy efficacy is inseparable from the stability of underlying chronic liver disease. The observation that liver function may deteriorate even without clear immune‐mediated injury is consistent with emerging hypotheses, suggesting that immunological, haemodynamic and metabolic factors may contribute to decompensation in susceptible patients. In our cohort, death without radiologic progression attributable to hepatic decompensation occurred in approximately 10% of the patients, indicating that while this outcome is clinically relevant, its overall incidence was not high. In this context, our CP evolution analysis offers complementary insight to findings from atezolizumab–bevacizumab studies, in which hepatic deterioration has also been recognised as a relevant clinical event [6, 7, 8, 9]. These data highlight the importance of monitoring and preserving the hepatic reserve during treatment [6]. Furthermore, the incidence of variceal bleeding in our cohort was low and the events occurred predominantly in patients with pre‐existing F2 varices, underscoring the importance of adequate baseline endoscopic assessment in appropriately managed patients with cirrhosis. Together, these data emphasise that preserving the hepatic reserve is not merely a supportive care goal but also a central therapeutic objective.

Beyond their biological interpretation, these results provide a practical framework for the clinical implementation of the STRIDE. The steep early hazard of toxicity suggests that the first 2 months of therapy represent a period of enhanced monitoring, during which timely recognition and management of both immune‐mediated and cirrhosis‐related events may influence long‐term outcomes. The relatively stable toxicity profile after this initial phase supports the feasibility of continued treatment in patients who navigate the early period without significant deterioration. Furthermore, the alignment of our efficacy estimates with those from other international real‐world experiences suggests that response assessment at standard intervals remains an adequate surrogate for identifying patients who are likely to derive prolonged benefits, an observation consistent with the strong prognostic impact of disease control across studies [15].

These findings have also shaped the research agenda of the field. The interplay between the tumour response, immune‐mediated toxicity and liver functional reserve calls for integrated models that can capture dynamic risk rather than static predictors. Incorporating serial CP trajectories, biomarkers of portal hypertension and potentially immune signatures could refine patient selection for dual checkpoint inhibition [19]. Moreover, interventions targeting cirrhosis, particularly optimisation of antiviral therapy, management of portal hypertension and nutritional or metabolic support, which may modulate both toxicity and survival, as suggested by recent evidence [12, 13, 20]. Understanding how these interventions interface with immunotherapy represents a promising direction for translational and clinical investigation.

Our findings should be interpreted in the context of emerging real‐world evidence on liver functional reserve and safety in advanced HCC, particularly in Child–Pugh B patients. A large Japanese multicentre study by Ohama et al. reported comparable survival outcomes between atezolizumab plus bevacizumab and durvalumab plus tremelimumab, but with distinct immune‐related toxicity profiles and differential ALBI score dynamics, highlighting the complexity of treatment selection in patients with borderline hepatic function [21]. In addition, real‐world data from Japan by Yonemoto et al. demonstrated the feasibility of durvalumab plus tremelimumab after prior atezolizumab plus bevacizumab, including rechallenge after immune‐related adverse events. In this context, our study complements the existing literature by providing a temporal characterisation of hepatic decompensation events and ALBI dynamics analysed alongside progression and death as competing outcomes within a regional real‐world cohort [22].

This study had several limitations that should be acknowledged. First, despite the prospective nature of the data collection, the analysis was retrospective, introducing the potential for selection bias and unmeasured confounders inherent to real‐world observational studies. Second, although the cohort reflects routine clinical practice, the sample size limits the power to detect modest differences and precludes robust multivariable modelling for some outcomes. In particular, some subgroups included in the Cox analyses (such as patients with ECOG performance status 2) were small, resulting in wide confidence intervals and potentially unstable hazard ratio estimates, which should be interpreted with caution.

Third, the median follow‐up of 8.9 months was relatively short, restricting the ability to fully characterise long‐term survival, durability of response and late‐onset irAEs. Fourth, radiological assessments were performed according to local practice rather than a centralised review, and heterogeneity in imaging schedules and criteria could introduce variability in response and PFS estimates. Finally, the limited number of high‐grade irAEs and early hepatic complications constrain the interpretation of hazard‐based toxicity patterns and the ability to identify reliable early surrogate markers of benefit or risk. Collectively, these limitations underscore the need for a longer follow‐up and larger prospective datasets to validate these preliminary findings. A further limitation is the partial overlap in authorship with other real‐world studies on durvalumab‐based regimens for advanced HCC. However, this analysis addressed a distinct research question, focusing on the temporal dynamics of toxicity and longitudinal liver function within a regional, prospectively curated cohort. The study populations, analytical approaches and endpoints differed from those in prior reports.

As the therapeutic landscape of unresectable HCC continues to expand, our study contributes to a nuanced picture of how STRIDE is performed in routine practice. The reproducible anti‐tumour activity, predictable concentration of toxicity in the early treatment period and central prognostic importance of liver reserve collectively strengthen the rationale for STRIDE as a frontline option. At the same time, they underscored that immunotherapy cannot be dissociated from the management of underlying liver disease. By illuminating these interdependencies, our findings offer both immediate clinical value and a foundation for refining therapeutic strategies aimed at maximising the benefits while preserving hepatic function.

## Author Contributions

Andrea Casadei‐Gardini, Massimo Iavarone and Lorenza Rimassa: conceptualisation, methodology, formal analysis, writing, original draft. Acquisition of data (acquired and managed patients): all authors.

## Funding

This Study was funded in part by ‘Ricerca Corrente’ (RC2026/105_01).

## Ethics Statement

The study was conducted in accordance with the Declaration of Helsinki and the protocol was approved by the Ethics Committee of each institution involved in the project. Under the condition of retrospective archival tissue collection and patients’ data anonymisation, our study was exempt from the acquisition of informed consent from patients by the institutional review board. Institutional Review Board Statement: The Ethical Review Board of each Institutional Hospital approved the present study. This study was performed in line with the principles of the Declaration of Helsinki.

## Consent

Written informed consent for treatment was obtained for all patients.

## Conflicts of Interest

Andrea Casadei‐Gardini received consulting fees from AbbVie, AstraZeneca, Bayer, BMS, Eisai, Incyte, Ipsen, Jazz Pharmaceuticals, MSD, Roche, Servier, Taiho Oncology; lecture fees from AstraZeneca, Bayer, BMS, Eisai, Incyte, Ipsen, Roche, Servier; travel expenses from AstraZeneca and Servier; and research grants (to Institution) from AstraZeneca and MSD. Federica Tosi received consulting fees from Astra Zeneca, Daiichi‐Sankyo. Salvatore Corallo reports consulting fees and/or speaker honoraria from Amgen, AstraZeneca, Merck. Serono, Daiichi‐Sankyo and travel grants from Amgen, AstraZeneca, MSD, Daiichi‐Sankyo and Roche. Gianluca Tomasello received consulting fees from Pfizer, Roche, Astra Zeneca, Istituto Gentili, Novartis and Servier; travel fees from Lilly, Novartis, Menarini, Sophos, Servier and Merck Serono. Tziana Pressiani reports consulting fees from Astra Zeneca and Roche, institutional research funding from Astra Zeneca, and support for congress attendance from Roche. Lorenza Rimassa received consulting fees from AbbVie, AstraZeneca, Basilea, Bayer, BMS, Boehringer Ingelheim, Eisai, Elevar Therapeutics, Exelixis, Genenta, Guerbet, Hengrui, Incyte, Ipsen, Jazz Pharmaceuticals, MSD, Nerviano Medical Sciences, Roche, Servier, Taiho Oncology, Zymeworks; lecture fees from AstraZeneca, Bayer, Biologix, BMS, Eisai, Guerbet, Incyte, Ipsen, Roche, Servier; travel expenses from AstraZeneca and Servier; research grants (to Institution) from AbbVie, AstraZeneca, BeiGene, Exelixis, Fibrogen, Incyte, Ipsen, Jazz Pharmaceuticals, MSD, Nerviano Medical Sciences, Roche, Servier, Taiho Oncology, TransThera Sciences, Zymeworks. Massimo Iavarone received consulting fees from Roche, Roche Diagnostics, EISAI, MSD and Astra Zeneca; lecture fees from Roche, Astra Zeneca, IPSEN, EISAI, MSD and Gilead; travel fees from Roche; and research founding (institution) from Gilead, Roche, Astra Zeneca and EISAI. All the other authors declares no conflicts of interest.

## Supporting information


**Figure S1:** Temporal hazard patterns of adverse events during STRIDE therapy.
Smoothed hazard functions depicting the timing of adverse events (AEs) from treatment initiation. Hazard rates are expressed as events per patient‐month and were estimated using kernel density methods.

**Figure S2:** Kaplan–Meier survival analyses according to radiological response.
(A) Overall survival (OS) stratified by objective response (complete response [CR] or partial response [PR] vs. stable disease [SD] or progressive disease [PD]). (B) Progression‐free survival (PFS) stratified by objective response (CR/PR vs. SD/PD). (C) Overall survival (OS) according to disease control (CR/PR/SD vs. PD). (D) Progression‐free survival (PFS) according to disease control (CR/PR/SD vs. PD).

**Figure S3:** Association between baseline clinical characteristics and radiological response. Forest plot showing the association between baseline variables and the probability of achieving objective response (CR/PR) at first radiological assessment.

**Table S1:** liv70640‐sup‐0004‐TableS1.docx.

**Table S2:** Summary of treatment‐related adverse events. Frequency and severity of treatment‐related adverse events (AEs) occurring during STRIDE therapy.Multivariable Cox regression analysis for overall survival.

**Table S3:** liv70640‐sup‐0006‐TableS3.docx.

## Data Availability

The data that support the findings of this study are available from the corresponding author upon reasonable request.
