# Supplementary figures and images for: Durvalumab–Tremelimumab in Advanced Hepatocellular Carcinoma: Real‐World Data From the LOR‐HCC (Lombardy Real‐World HCC Group)
Source: Liver Int. 2026 Apr 16;46(5):e70640. doi: 10.1111/liv.70640 (PMC13087547; doi:10.1111/liv.70640)

## Slide 1
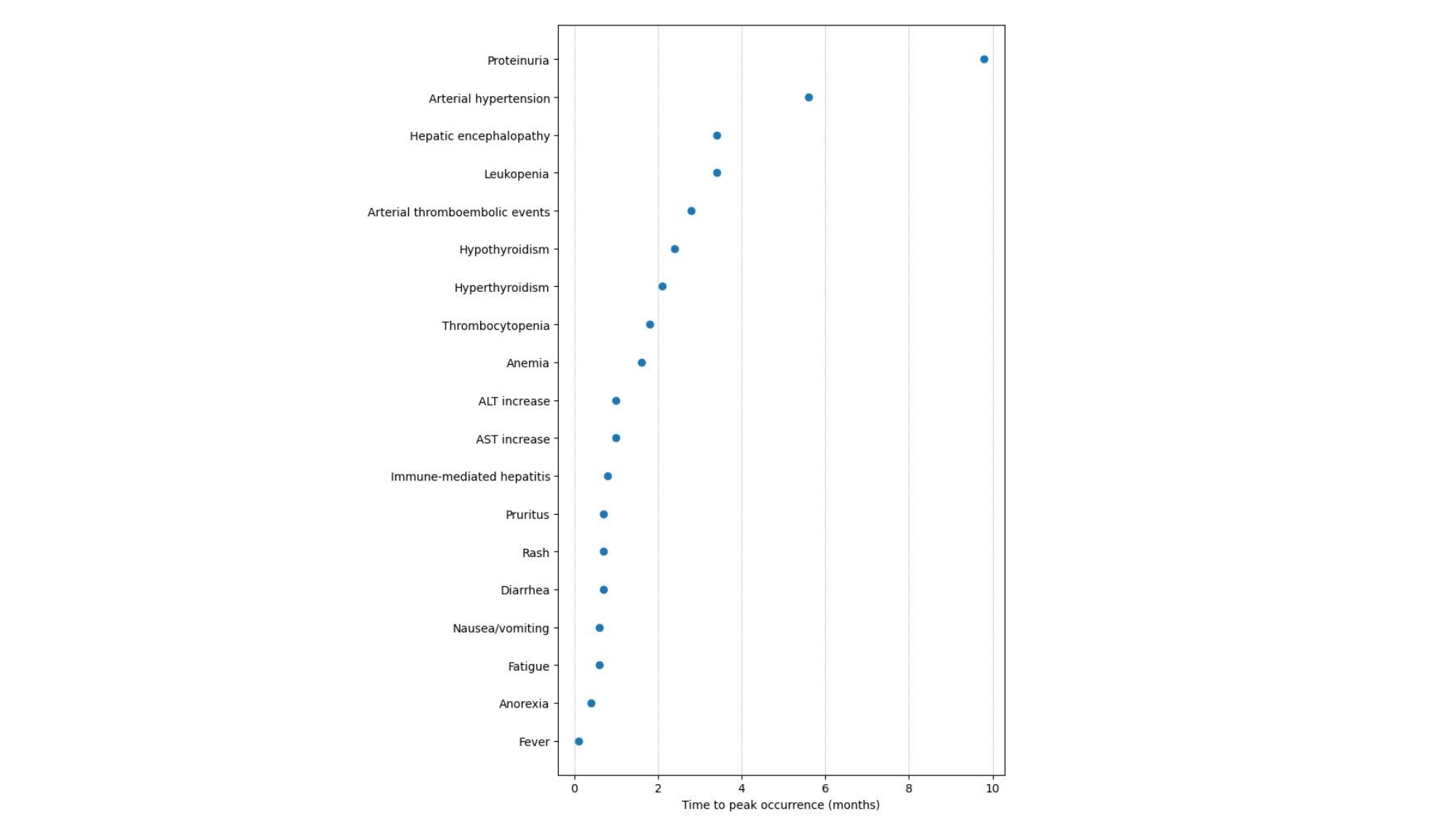

#

Supplement: Supplementary file 1 — Figure S1: Temporal hazard patterns of adverse events during STRIDE therapy. Smoothed hazard functions depicting the timing of adverse events (AEs) from treatment initiation. Hazard rates are expressed as events per patient‐month and were estimated using kernel density methods. [file LIV-46-0-s004.pptx]

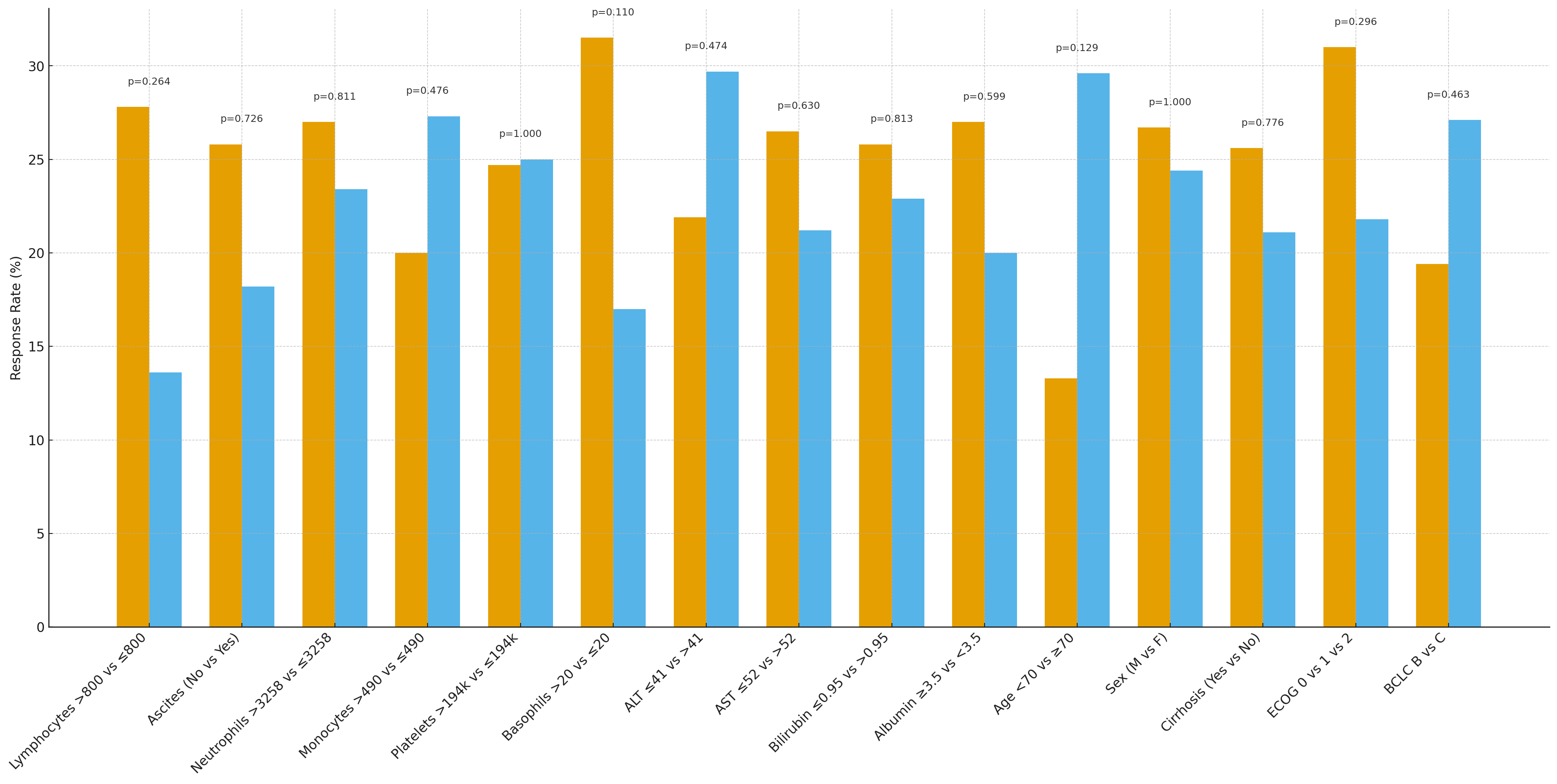

Supplement: Supplementary file 3 — Figure S3: Association between baseline clinical characteristics and radiological response. Forest plot showing the association between baseline variables and the probability of achieving objective response (CR/PR) at first radiological assessment. [file LIV-46-0-s006.png]
