# Supplementary material for: Durvalumab–Tremelimumab in Advanced Hepatocellular Carcinoma: Real‐World Data From the LOR‐HCC (Lombardy Real‐World HCC Group)
Source: Liver Int. 2026 Apr 16;46(5):e70640. doi: 10.1111/liv.70640 (PMC13087547; doi:10.1111/liv.70640)
Supplement: Supplementary file 2 — Figure S2: Kaplan–Meier survival analyses according to radiological response. (A) Overall survival (OS) stratified by objective response (complete response [CR] or partial response [PR] vs. stable disease [SD] or progressive disease [PD]). (B) Progression‐free survival (PFS) stratified by objective response (CR/PR vs. SD/PD). (C) Overall survival (OS) according to disease control (CR/PR/SD vs. PD). (D) Progression‐free survival (PFS) according to disease control (CR/PR/SD vs. PD). [file LIV-46-0-s003.pptx]

## Slide 1
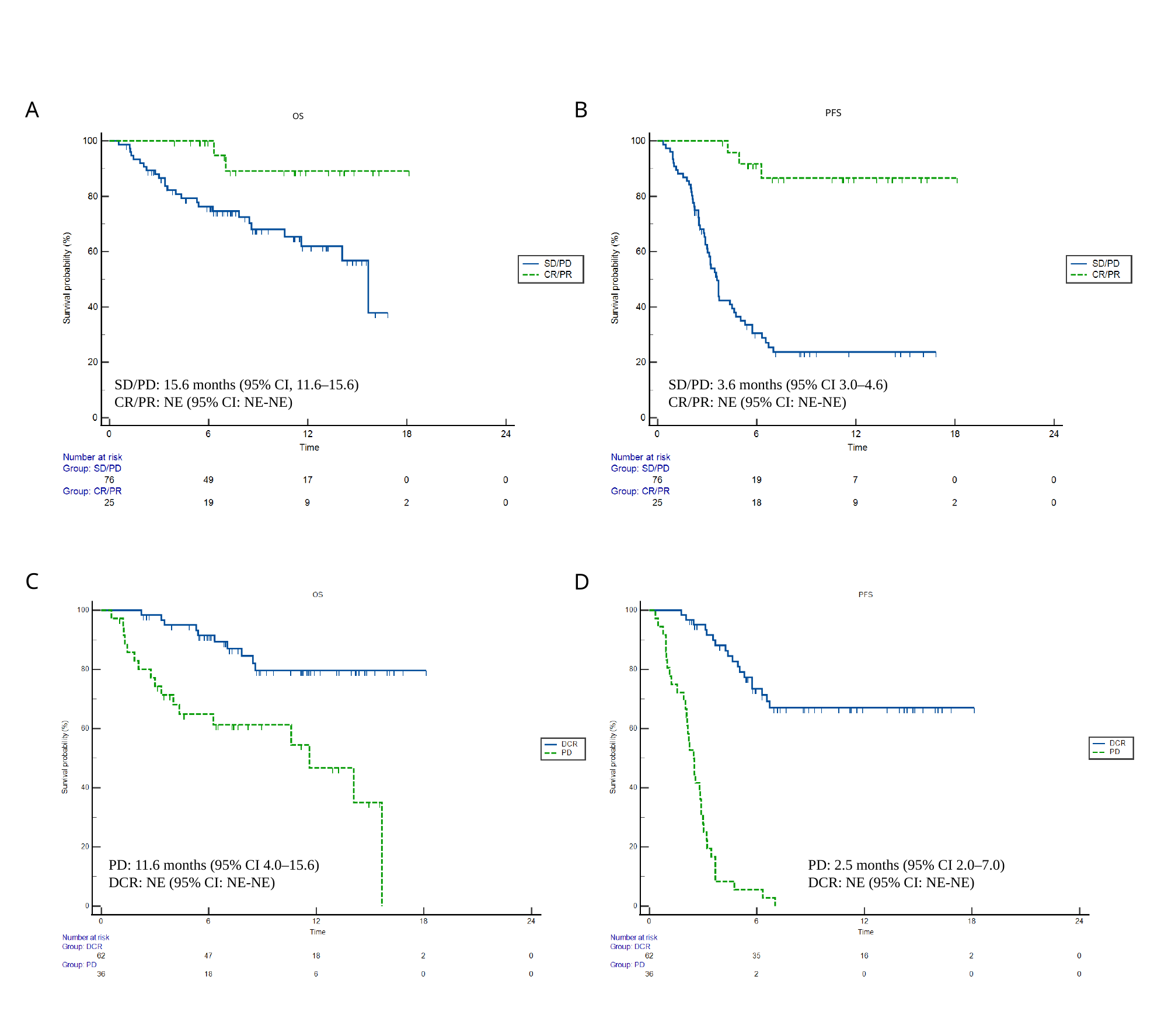

A
B
PFS
OS
SD/PD: 3.6 months (95% CI 3.0–4.6)
CR/PR: NE (95% CI: NE-NE)
SD/PD: 15.6 months (95% CI, 11.6–15.6)
CR/PR: NE (95% CI: NE-NE)
C
D
PD: 11.6 months (95% CI 4.0–15.6)
DCR: NE (95% CI: NE-NE)
PD: 2.5 months (95% CI 2.0–7.0)
DCR: NE (95% CI: NE-NE)
