# Supplementary material for: Durvalumab–Tremelimumab in Advanced Hepatocellular Carcinoma: Real‐World Data From the LOR‐HCC (Lombardy Real‐World HCC Group)
Source: Liver Int. 2026 Apr 16;46(5):e70640. doi: 10.1111/liv.70640 (PMC13087547; doi:10.1111/liv.70640)
Supplement: Supplementary file 4 — Table S1: liv70640‐sup‐0004‐TableS1.docx. [file LIV-46-0-s001.docx]

Supplementary Table 1.

| Adverse Event | Patients with AEs (>0, %) | Patients without AEs (=0, %) | Grade 0–2 (%) | Grade >2 (%) |
| --- | --- | --- | --- | --- |
| Fatigue | 37.4 | 62.6 | 95.7 | 4.3 |
| Skin rash | 30.4 | 69.6 | 96.5 | 3.5 |
| Itching | 29.6 | 70.4 | 98.3 | 1.7 |
| AST increase | 18.0 | 82.0 | 94.6 | 5.4 |
| ALT increase | 17.1 | 82.9 | 95.5 | 4.5 |
| Ascites | 16.2 | 83.8 | 92.8 | 7.2 |
| Fever | 13.9 | 86.1 | 100.0 | 0.0 |
| Diarrhea | 12.3 | 87.7 | 96.5 | 3.5 |
| Bilirubin increase | 11.7 | 88.3 | 98.2 | 1.8 |
| Hypothyroidism | 10.8 | 89.2 | 98.2 | 1.8 |
| Anorexia | 9.6 | 90.4 | 99.1 | 0.9 |
| Thrombocytopenia | 7.8 | 92.2 | 99.1 | 0.9 |
| Immune-mediated hepatitis | 7.2 | 92.8 | 98.2 | 1.8 |
| Anemia | 7.0 | 93.0 | 98.3 | 1.7 |
| Immune-mediated pneumonia | 6.3 | 93.7 | 99.1 | 0.9 |
| Abdominal pain | 5.5 | 94.5 | 99.1 | 0.9 |
| Nausea/vomiting | 5.3 | 94.7 | 99.1 | 0.9 |
| Hypethyroidism | 4.5 | 95.5 | 100.0 | 0.0 |
| Immune-mediated colitis | 4.5 | 95.5 | 96.4 | 3.6 |
| Weight loss | 4.3 | 95.7 | 100.0 | 0.0 |
| E/G varices | 3.6 | 96.4 | 97.3 | 2.7 |
| Hepatic encephalopathy | 2.7 | 97.3 | 98.2 | 1.8 |
| Arterial hypertension | 2.7 | 97.3 | 98.2 | 1.8 |
| Leucopenia | 2.6 | 97.4 | 98.3 | 1.7 |
| Acute heart failure | 1.8 | 98.2 | 99.1 | 0.9 |
| Other digestive hemorrhage | 1.8 | 98.2 | 99.1 | 0.9 |
| Mucositis/Dysgeusia | 0.9 | 99.1 | 100.0 | 0.0 |
| Arterial thromboembolic events | 0.9 | 99.1 | 99.1 | 0.9 |
| Epistaxis | 0.9 | 99.1 | 100.0 | 0.0 |
| Proteinuria | 0.9 | 99.1 | 100.0 | 0.0 |
