# Supplementary material for: Durvalumab–Tremelimumab in Advanced Hepatocellular Carcinoma: Real‐World Data From the LOR‐HCC (Lombardy Real‐World HCC Group)
Source: Liver Int. 2026 Apr 16;46(5):e70640. doi: 10.1111/liv.70640 (PMC13087547; doi:10.1111/liv.70640)
Supplement: Supplementary file 5 — Table S2: Summary of treatment‐related adverse events. Frequency and severity of treatment‐related adverse events (AEs) occurring during STRIDE therapy.Multivariable Cox regression analysis for overall survival. [file LIV-46-0-s002.docx]

# Supplementary Table 2.

| **Domain / Variable** | **Category** | **Univariate**  **HR (95% CI)** | **Univariate**  **p-value** | **Multivariate**  **HR (95% CI)** | **Multivariate**  **p-value** |
| --- | --- | --- | --- | --- | --- |
| Demographics | Age ≥70 vs <70 | 0.76 (0.35–1.63) | 0.48 | — |  |
|  | Female vs male | 0.18 (0.05–0.57) | 0.0039 | 0.35 (0.14–0.90) | 0.028 |
| Tumor-related | AFP ≥400 vs <400 | 1.21 (0.52–2.84) | 0.65 | — |  |
|  | BCLC C vs A/B | 2.11 (1.03–4.35) | 0.041 | 1.64 (0.62–4.35) | 0.31 |
|  | Extrahepatic spread  (yes vs no) | 2.27 (1.10–4.55) | 0.025 | — |  |
|  | Portal vein thrombosis  (yes vs no) | 1.03 (0.49–2.19) | 0.93 | — |  |
| Liver function | Albumin  >3.5 vs <3.5 g/dL | 0.22 (0.09–0.54) | 0.0011 | 0.65 (0.30–1.46) | 0.31 |
|  | Bilirubin  above normal vs normal | 2.26 (1.09–4.69) | 0.028 | 1.55 (0.72–3.36) | 0.26 |
|  | Child–Pugh  B vs A | 2.00 (0.61–6.49) | 0.25 | — |  |
|  | ALBI grade  >1 vs 1 | 0.68 (0.21–2.24) | 0.52 | — |  |
|  | Cirrhosis  (yes vs no) | 0.95 (0.41–2.23) | 0.91 | — |  |
|  | Ascites  (yes vs no) | 2.29 (0.68–7.76) | 0.18 | — |  |
| Laboratory parameters | ALT  >41 vs ≤41 U/L | 2.77 (1.33–5.78) | 0.0063 | 1.06 (0.43–2.59) | 0.89 |
|  | AST  ≥52 vs <52 U/L | 4.85 (2.19–10.7) | 0.0001 | 3.01 (1.22–7.40) | 0.016 |
|  | Creatinine  ≥1.72 vs <1.72 mg/dL | 1.07 (0.31–3.64) | 0.91 | — |  |
|  | Hemoglobin  ≥9.9 vs <9.9 g/dL | 0.62 (0.11–3.43) | 0.58 | — |  |
|  | Platelets  ≥194 vs <194 ×10³/µL | 2.11 (0.94–4.76) | 0.07 | — |  |
|  | Sodium  ≥135 vs <135 mmol/L | 0.21 (0.06–0.77) | 0.018 | 0.86 (0.36–2.07) | 0.74 |
| Inflammatory cells | Neutrophils  ≥3200 vs <3200 /µL | 1.56 (0.77–3.17) | 0.22 | — |  |
|  | Lymphocytes  ≥800 vs <800 /µL | 1.51 (0.66–3.45) | 0.32 | — |  |
|  | Monocytes  ≥490 vs <490 /µL | 1.92 (0.94–3.92) | 0.07 | — |  |
|  | Eosinophils  ≥120 vs <120 /µL | 1.21 (0.61–2.40) | 0.59 | — |  |
|  | Basophils  ≥20 vs <20 /µL | 1.56 (0.78–3.12) | 0.20 | — |  |
| Performance status | ECOG PS  1 vs 0 | 2.49 (1.25–4.97) | 0.0032 | 2.95 (1.17–7.40) | 0.02 |
|  | ECOG PS  2 vs 0 | 7.16 (0.75–67.8) | — | 5.95 (1.27–27.9) | 0.02 |
| Etiology | ALD  (yes vs no) | 1.44 (0.58–3.55) | 0.43 | — |  |
|  | HBV  (yes vs no) | 0.73 (0.30–1.74) | 0.48 | — |  |
|  | HCV  (yes vs no) | 1.49 (0.74–2.99) | 0.26 | — |  |
|  | MASLD  (yes vs no) | 1.00 (0.47–2.11) | 0.99 | — |  |
|  | Met-ALD  (yes vs no) | 0.99 (0.34–2.84) | 0.99 | — |  |
