# Supplementary material for: Durvalumab–Tremelimumab in Advanced Hepatocellular Carcinoma: Real‐World Data From the LOR‐HCC (Lombardy Real‐World HCC Group)
Source: Liver Int. 2026 Apr 16;46(5):e70640. doi: 10.1111/liv.70640 (PMC13087547; doi:10.1111/liv.70640)
Supplement: Supplementary file 6 — Table S3: liv70640‐sup‐0006‐TableS3.docx. [file LIV-46-0-s005.docx]

# Supplementary Table 3.

| **Domain / Variable** | **Category** | **Univariate HR**  **(95% CI)** | **Univariate**  **p-value** | **Multivariate HR (95% CI)** | **Multivariate**  **p-value** |
| --- | --- | --- | --- | --- | --- |
| Demographics | Age  ≥70 vs <70 | 0.77 (0.43–1.40) | 0.39 | — |  |
|  | Female vs male | 0.58 (0.26–1.29) | 0.18 | — |  |
| Tumor-related | AFP  >400 vs ≤400 | 1.42 (0.74–2.71) | 0.29 | — |  |
|  | BCLC  C vs A/B | 1.54 (0.89–2.64) | 0.12 | — |  |
|  | Extrahepatic spread  (yes vs no) | 1.69 (0.98–2.92) | 0.06 | — |  |
|  | Portal vein thrombosis  (yes vs no) | 0.58 (0.32–1.03) | 0.06 | — |  |
| Liver function | Albumin  >3.5 vs ≤3.5 g/dL | 0.55 (0.28–1.07) | 0.08 | — |  |
|  | Child–Pugh  B vs A | 4.01 (1.51–10.7) | 0.0054 | 2.20  (1.08–4.46) | 0.028 |
|  | ALBI grade  >1 vs 1 | 0.84 (0.32–2.16) | 0.71 | — |  |
|  | Cirrhosis  (yes vs no) | 0.90 (0.48–1.72) | 0.76 | — |  |
|  | Ascites  (yes vs no) | 1.83 (0.74–4.52) | 0.19 | — |  |
| Laboratory parameters | ALT  >41 vs ≤41 U/L | 1.42 (0.81–2.47) | 0.21 | — |  |
|  | AST  >52 vs ≤52 U/L | 2.40 (1.29–4.46) | 0.0056 | 2.22  (1.28–3.87) | 0.0046 |
|  | Total bilirubin  above normal vs normal | 1.13 (0.65–1.97) | 0.67 | — |  |
|  | Creatinine  >1.72 vs ≤1.72 mg/dL | 0.84 (0.33–2.16) | 0.72 | — |  |
|  | Hemoglobin  >9.9 vs ≤9.9 g/dL | 0.27 (0.07–1.03) | 0.055 | — |  |
|  | Platelets  >194 vs ≤194 ×10³/µL | 1.11 (0.59–2.06) | 0.75 | — |  |
|  | Serum sodium  >135 vs ≤135 mmol/L | 0.41 (0.14–1.16) | 0.09 | — |  |
| Inflammatory cells | Neutrophils  >3200 vs ≤3200 /µL | 1.05 (0.61–1.80) | 0.87 | — |  |
|  | Lymphocytes  >800 vs ≤800 /µL | 1.11 (0.58–2.10) | 0.75 | — |  |
|  | Monocytes  >490 vs ≤490 /µL | 0.99 (0.57–1.71) | 0.97 | — |  |
|  | Eosinophils  >120 vs ≤120 /µL | 1.17 (0.69–1.96) | 0.56 | — |  |
|  | Basophils  >20 vs ≤20 /µL | 0.72 (0.42–1.22) | 0.22 | — |  |
| Performance status | ECOG PS  1 vs 0 | 2.15 (1.26–3.65) | 0.02 | 2.38  (1.32–4.29) | 0.0039 |
|  | ECOG  PS 2 vs 0 | 0.82 (0.19–3.58) | — | 1.45 (1.03–3.56) | 0.0039 |
| Etiology | ALD  (yes vs no) | 1.58 (0.81–3.05) | 0.17 | — |  |
|  | HBV  (yes vs no) | 1.37 (0.72–2.60) | 0.33 | — |  |
|  | HCV  (yes vs no) | 1.12 (0.65–1.91) | 0.69 | — |  |
|  | MASLD  (yes vs no) | 0.64 (0.36–1.14) | 0.13 | — |  |
|  | Met-ALD  (yes vs no) | 1.11 (0.49–2.50) | 0.80 | — |  |
